# Novel pH-responsive nanohybrid for simultaneous delivery of doxorubicin and paclitaxel: an in-silico insight

**DOI:** 10.1186/s13065-021-00735-4

**Published:** 2021-02-11

**Authors:** Ehsan Alimohammadi, Reza Maleki, Hossein Akbarialiabad, Mohammad Dahri

**Affiliations:** 1 Neurosurgery Department, Kermanshah University of Medical Sciences, Kermanshah, Iran; 2 Computational Biology and Chemistry Group (CBCG), Universal Scientific and Education and Research Network (USERN), Tehran, Iran; 3 Student Research Committee, School of Medicine, Shiraz University of Medical Sciences, Shiraz, Iran; 4 Student Research Committee, School of Pharmacy, Shiraz University of Medical Sciences, Shiraz, Iran

**Keywords:** Doxorubicin, Paclitaxel, Molecular dynamics, Drug release, Smart drug delivery, Fullerene

## Abstract

**Background:**

The distribution of drugs could not be controlled in the conventional delivery systems. This has led to the developing of a specific nanoparticle-based delivery system, called smart drug delivery systems. In cancer therapy, innovative biocompatible nanocarriers have received much attention for various ranges of anti-cancer drugs. In this work, the effect of an interesting and novel copolymer named "dimethyl acrylamide-trimethyl chitosan" was investigated on delivery of paclitaxel and doxorubicin applying carboxylated fullerene nanohybrid. The current study was run via molecular dynamics simulation and quantum calculations based on the acidic pH differences between cancerous microenvironment and normal tissues. Furthermore, hydrogen bonds, radius of gyration, and nanoparticle interaction energies were studied here. Stimulatingly, a simultaneous pH and temperature-responsive system were proposed for paclitaxel and doxorubicin for a co-polymer. A pH-responsive and thermal responsive copolymer were utilized based on trimethyl chitosan and dimethyl acrylamide, respectively. In such a dualistic approach, co-polymer makes an excellent system to possess two simultaneous properties in one bio-polymer.

**Results:**

The simulation results proposed dramatic and indisputable effects of the copolymer in the release of drugs in cancerous tissues, as well as increased biocompatibility and drug uptake in healthy tissues. Repeated simulations of a similar article performed for the validation test. The results are very close to those of the reference paper.

**Conclusions:**

Overall, conjugated modified fullerene and dimethyl acrylamide-trimethyl chitosan (DMAA-TMC) as nanohybrid can be an appropriate proposition for drug loading, drug delivery, and drug release on dual responsive smart drug delivery system.
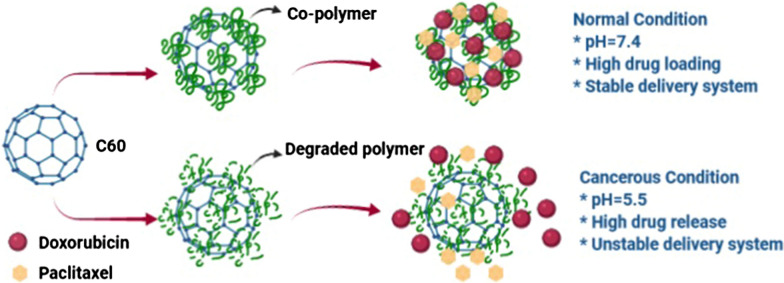

## Introduction

Multiple factors are involved in cancer initiation and then in progression mechanisms [1]. These factors can be intrinsic, such as susceptibility to Chronic myelogenous leukemia (CML) in a newborn with Philadelphia Chromosome or extrinsic, including tobacco smoking, diet, infectious disease, ion, and non-ion radiation, and so on (2–9). Nowadays, various treatment methods, including chemotherapy, radiotherapy, and target-therapy, are common ways of cancer therapy. Each of them has specific pros and cons. Researchers are focusing on optimizing the drug’s efficacy while reducing undesired side effects [10–12]. Currently, nanobiotechnology has gained ground in cancer treatment and diagnosis [13–15]. Development in targeted nano-drug delivery has resulted in the progression of the smart drug delivery concept [16–19]. Such a system can increase the therapeutic index and drug concentration in the affected tissue and reduce the damage to healthy cells [20].

Doxorubicin and paclitaxel (PAX) are two crucial common combinations of anticancer agents in cancer therapy. Previous simultaneous administration studies suggested that DOX/PAX combination could be useful in metastatic cancer, especially in breast cancer. Due to synergism, this combination effectively inhibits multidrug resistance (MDR) cancer cells. As an anthracycline, DOX interferes and interacts with the synthesizing of essential proteins for neoplastic cell proliferation and impede cancer growth [21–23]. It seems that this drug acts via binding to DNA and subsequent inhibition of nucleic acid production by disrupting the molecular structure and further steric hindrance [24]. As with any anti-cancer drug, one of the undesirable side effects can be damaging surrounding cells of the tumor [25, 26]. As mentioned above, targeted drug delivery can minimize this drug's impacts on the body's non-cancerous cells and maximize the drug efficiency for cancer treatment [22, 27–30]. PAX is a hydrophobic antimicrotubular agent that inhibits microtubules' synthesizing from tubulin dimers and stabilizes the microtubules by preventing depolymerization [28, 31]. This stabilization inhibits the dynamic identification of the microtubule network required for critical interphase and mitotic cellular functions [32, 33]. PAX is one of the most effective drugs in the treatment of breast cancer with chemotherapy. Also, utilizing nanocarriers to optimize PAX’s efficacy seems to be promising [34].

Nano-based Drug delivery systems have an essential role in developing chemotherapeutic agents, cancer drug targeting, the selective antiproliferative effect [35, 36], and minimizing adverse drug side-effects [37, 38]. Multiple nanoparticles are capable carriers in enhancing the delivery of DOX/PAX; these agents are solid lipid nanoparticles [39]. Among these, 60-carbon modified fullerenes (C60) have recently received much attention in DOX/PAX drug co-delivery. The C60s are about 1 nm in diameter, nearly half the width of a DNA helix; it has a small size and spherical shape and conveniently crosses biological membranes and barriers and reach the cell [40]. These particles' surface properties can be readily modified and functionalized using functional groups and compounds by having extended surfaces area. The functionalization of these particles will increase their solubility, biocompatibility, and potential to deliver various materials within the body. These particles can be used as carriers of biological molecules such as protein, DNA, and drugs. Therapeutic compounds could be loaded onto these nanostructures [41]. Furthermore, another fascinating topic in drug delivery is co-targeting and co-transportation, in which two or more compounds are targeted and transported. Many surveys have studied the potential of fullerene [42]. The particular physical and chemical features of C60 as a nanocarrier for anticancer agents for drug delivery include size, triangular shape, surface charge, surface chemistry, hydrophobicity, loading’s potential, and especially the ability to cross various biological barriers in vivo without making an immune response.

Evidence is scarce concerning the potential toxicity of C60 in the human body compared to other nanomaterials [43]. The possible pathways in uptaking, distributing, metabolizing, and excreting nC60 are not transparent yet [44]. Pristine C60 seems to exert antioxidant activity as a free radical scavenger [45]. The reports about the possible toxicities are controversial. Some believe that at least in physiological conditions, it seems to have no or minimal acute or subacute adverse effect both in-vitro and in-vivo [46, 47]. An in-vitro study by Prylutska et al. showed that Nano-C60 accumulation in aqueous water did not have a toxic impact on lymphocytes’ genome [47–50]. An in vivo study in mice showed that the toxicity of c60 in the aqueous colloid solution could be noticed in a dose-dependent manner. It showed no adverse effect at low doses, and at the higher dosed nervous system, hematologic system, and other systems were impaired. The authors recommended the non-toxic dose for biomedical application of the C60 is 75–150 mg/kg [47]. Another study showed that the mice had an inflammatory pulmonary response subsequent to inhalation of the C60 [51]. Vasyukova et al. showed that C60 harms the embryo of mammalians. Although it is not still transparent, it seems that the possible pathways for inducing the cytotoxicity are interfering with the metabolism of tetrahydrofuran (THF), leading to its decomposition [49, 50].

C60 can bond with specific cell receptors and intracellular target molecules for targeted delivery of therapeutic agents [52]. Research has shown that C60 enters the cell vertically through a mediated endocytosis mechanism because of clathrin similarity. The drug can be encapsulated by the C60 and protected during circulation through the body. After reaching the target site, the encapsulating materials will be degraded after the drug release from the C60. The encapsulated drug should be proportional to the diameter and size of the C60 [53]. Despite their specific inherent properties, relevant concerns have been mentioned regarding the toxicity of C60, as several studies have manifested that pristine C60 can instigate biological destruction [53].

In this work, C60 bioconjugation with a novel biodegradable and biocompatible polymer dispels its biological concerns and converts it to a safer, more reliable nanocarrier. On the other hand, many investigations indicated that the C60 functionalized by a carboxylic group rendered it pharmacodynamically and pharmacokinetically better for drug delivery. Overall, the pH-sensitive C60 modified with non-bonded interaction of dimethyl acryl amide-trimethyl chitosan (DMAA-TMC) in this work produces a biocompatible C60 that can capably be nominated as smart and target delivery systems. DMAA-TMC, as a modified polysaccharide from chitosan, plays a vital role in the adsorption enhancement of novel macromolecule delivery systems. In previous studies, chitosan and acrylamide polymers' role was clarified separately to improve drug delivery systems. In our current study, the combination of two polymers DMAA and TMC, in-sillico by C60 carrier was investigated for the first time. Moreover, it’s non-bonded interaction with C60 and positive surface charge induce the specific properties in drug adsorption and drug release. Because of pores in DMAA-TMC, modified C60 was evaluated as a potential tool to improve the loading and co-release of DOX/PAX in physiological and cancerous pH, respectively [54].

Verma et al. investigated the loading of DOX on chitosan. They found that binding the dox with chitosan has facilitated the distribution of DOX in different organs of mice. The positive charge of chitosan and the negative charge of cell membrane or proteins helped to dox to interfere with DNA [55, 56]. 100% of treated mice by DOX-chitosan survived. Mai et al. designed the combination of trimethyl chitosan with a copolymer to investigated the co-delivery of DOX and iSur-pDNA. [57]. This pH-responsive sustained-release system possessed desired in vivo safety. This work indicated that the co-delivery of therapeutic compounds are more effective than single therapy.

Molecular dynamics is a powerful tool that can provide qualitative and quantitative information on pharmaceutical systems' physicochemical interactions and mechanisms. In the study of molecular dynamics, a system is first considered that consists of “N” particles inside a box called “simulation box” The particles' location and velocity at each step can be used to calculate all the system's static and dynamic properties. From the theorists' point of view, the importance of molecular dynamics studies is that they provide accurate quasi-experimental results for a well-defined model. Molecular dynamics serves somewhere between laboratory experiments and theory and is considered a virtual analysis [20].

Empirical experiments are fundamental but may impose a significant financial burden on researchers. Hence, there are several studies on MD to model delivery systems in cancer. However, therefore, the C60s as an attractive carrier for drug release were used to compare the uptake, diffusion, and release of DOX and PAX from C60 in the presence of chitosan polymer. Given the unique properties of C60, this could be an excellent introduction to the broader use of carbon C60 in the loading and release of anticancer drugs [58, 59].

## Method

### Molecular dynamics simulation

GROMACS 5.1.2 software was used to perform the simulation; the input structures were prepared with the OPLS-aa force field. Using the ACPYPE script, the parameters of the molecule were converted to GROMACS format. All the particles were placed inside the box, and the TIP3P water model was used as the solvent [60]. In the next step, the system temperature gradually increased from 0 to 310 K for 100 picoseconds in constant volume, using the Nose–Hoover algorithm [61, 62].

Moreover, the temperature system coupling rate of 0.5 ps was used, and then at the constant pressure was equilibrated for 200 ps. We used the Parrinello-Rahman algorithm to balance the system pressure. Molecular dynamics simulation was performed at 37° C for 50 ns. The cut-off distance was set at 1.2 Particle mesh Ewald applied to compute the electrostatic energy. The LINCS algorithm was performed to maintain the length of all links. To increase computational speed, the SHAKE algorithm was used to limit the hydrogen atom’s bonds.

### Carbon nanostructures parameters

The carbon atom charge in these nanostructures was assumed based on the use of naphthalene structure in the zero oplsaa force field. The types of bonds between carbon atoms were defined based on amino acids phenylalanine, tyrosine, and tryptophan [63]. The angle type is also determined based on the angles of the aromatic amino acid phenylalanine ring. The charge and the functional groups' parameters on this nanostructure were defined using a similar structure existing in the oplsaa force field. Lenard-Jones models and Colombian potentials were used to calculate non-bonding interactions such as electrostatic and van der Waals, respectively.

### The acidic and neutral condition

Calculations were performed to simulate the acidic and neutral states according to the following points: [1] The molecules of doxorubicin, paclitaxel, and DMAA-TMC and c60 polymers modified by carboxylic acid groups have different charges under neutral and acidic conditions. [2] First, two modes for each molecule were designed according to their charge in acidic and neutral conditions by Avogadro software. Then these molecules were optimized by Gaussian 09 software (B3LYP method and based set, 6–311 +  + G *). We calculated their charges through esp population using Gaussian software. Then The molecules were parameterized using the x2top command in GROMACS. Additional molecule information was also calculated using the OBGMX server. [3] The charge of carrier and DOX are indicated in Fig. 5.

### Quantum modeling

Two simulations were run by the DFT method utilizing Gaussian software. The structure of C60 is functionalized with a carboxyl group. And doxorubicin charged positively with protons at the amine group as an acidic state. Single point energy modeling was performed with the B3LYP method and with Basis Set, 6–311 + G *. At the end, the two adsorptions energies between C60 and DOX extracted.

## Results and discussion

To predict the co-adsorption of DOX/PTX on the DMAA-TMC functionalized C60, utilizing various computer-based mechanisms, co-release and aggregation of drugs were investigated in cancerous and physiological conditions with pHs 5.5 and 7.4, respectively.

### Simulation equilibrium

**Fig. 1:**
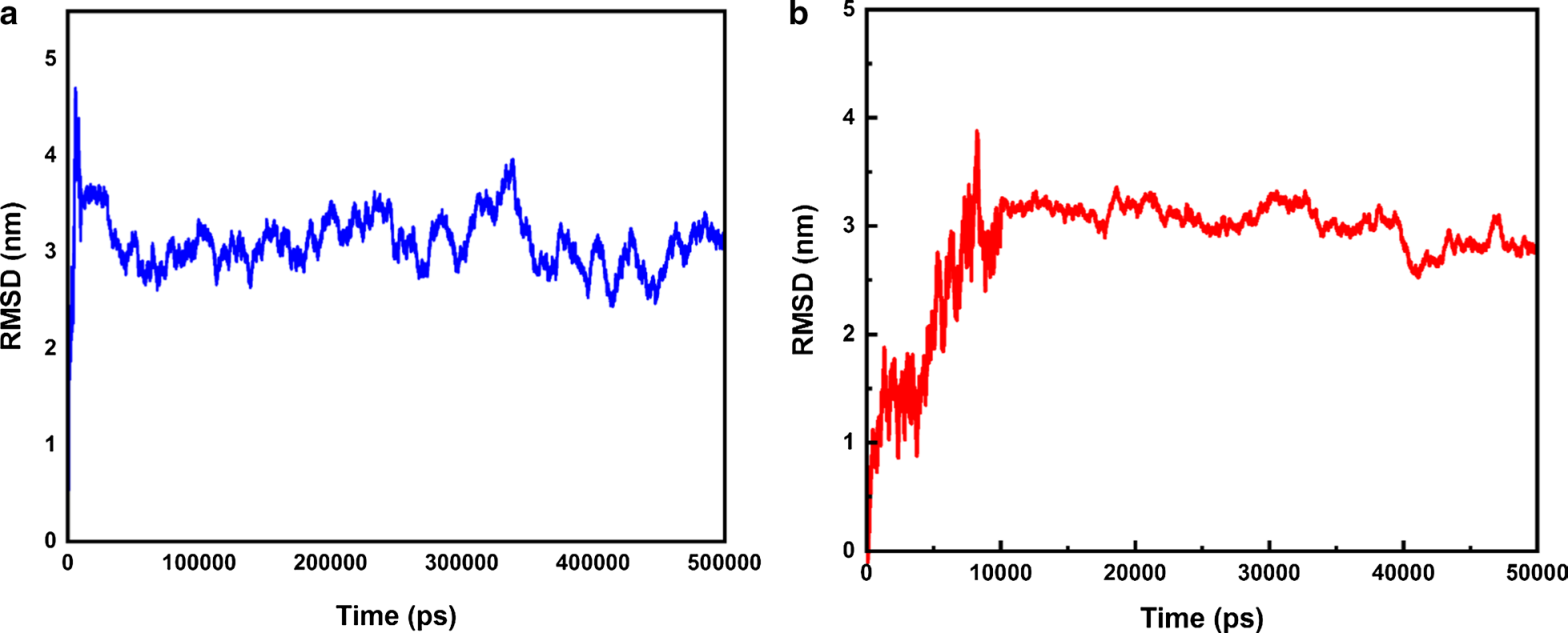
**a** RMSD of simulation system during 500 ns. **b** RMSD of simulation system during 50 ns

Root mean square deviation (RMSD) is a computational factor for the evaluation of the maintenance of integrity and balance of DDS during the simulation. In Fig. 1a, the simulation was performed throughout 500 ns. The slope of the variation vs time in less than 50 ns is about zero. The maximum RMSD values for 50 ns and 500 ns are 3.10 nm and 2.83 nm, respectively (Fig. 1a, b). Therefore, a duration of 50 ns is a reasonable and appropriate time to reach equilibrium and perform simulations.

### Particles accumulation and aggregation

**Fig. 2:**
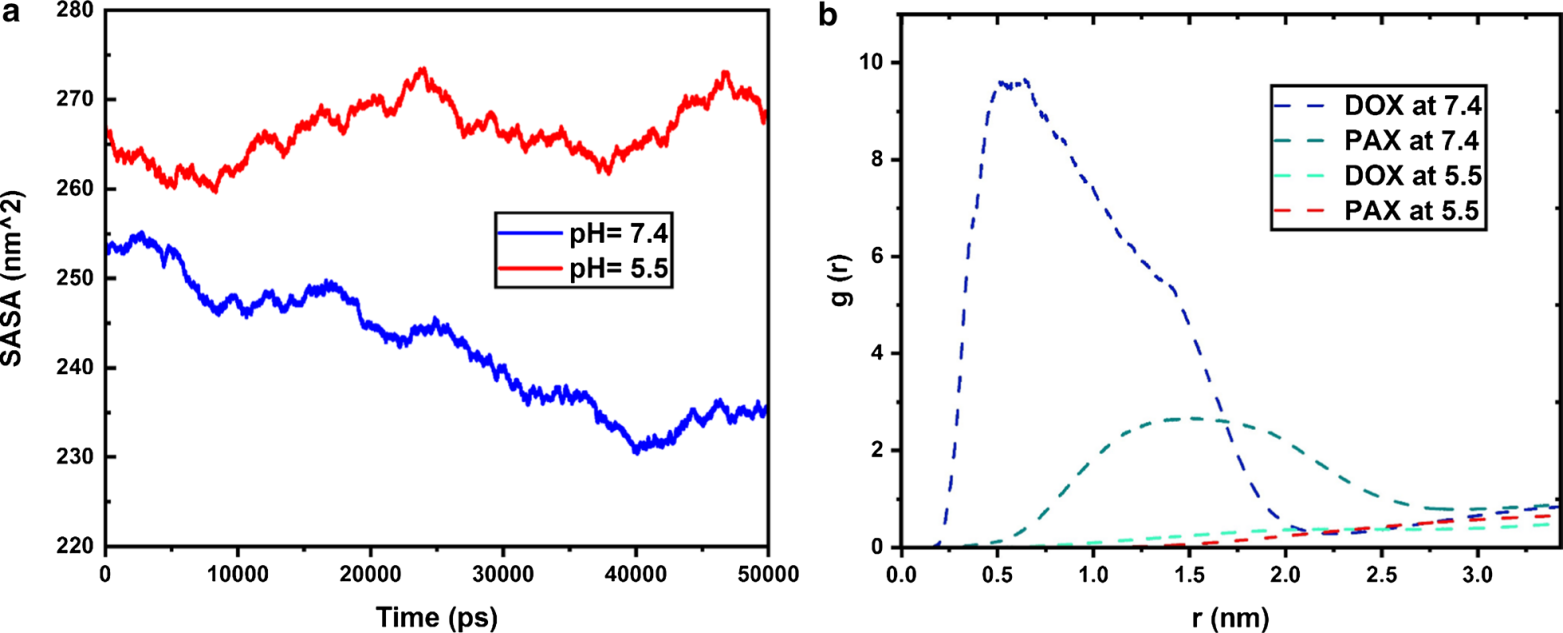
**a** SASA around C60. During 50 ns at two different pHs. **b** Radial distribution function around DOX and PAX at two different pHs

To evaluate the contact surface of the C60 with water, solvent accessible surface areas analysis was performed at both acidic and neutral states. According to Fig. 2a, the higher sass is for acidic pH and the lower sass is for neutral. This analysis is for the C60. Illustrations show that the contact surface of the C60 with water is higher and SASA is lower, in the neutral state. Therefore, the drugs and co-polymer are closer to the carrier in the neutral state. The opposite assertion is also true for the acidic state. The average of SASA during 50 ns for neutral and acidic pHs are 242.26 nm^2^ and 266.37 nm^2^, respectively (Fig. 2a).

The RDF (radial distribution functions) parameter can be used to investigate molecular aggregation in the simulation box. This analysis was performed by the gmx rdf command. The higher value, the greater and more stable the molecular aggregation. This parameter can also indicate drug loading. The higher the RDF, the higher the loading along with polymers and carriers. As shown below figure the RDF illustration indicates the aggregation of particles in a specific location of the simulation box. At neutral conditions, doxorubicin is absorbed by the carrier and the polymer. Doxorubicin has the highest value in the chart. Therefore, a neutral environment (the general blood environment of the body) has a proper load of the drug. But in an acidic environment, as you can see, it has less value. This indicates less accumulation of particles in the cancerous environment. The difference is that because the energies between PAX and the polymer and the carrier are less than DOX, the amount is also lower in the neutral medium. On the other hand, the same analysis applies to paclitaxel. However, energies between PAX and the polymer and the carrier are less than DOX, so the amount is less in a neutral environment (Fig. 2b).

### Drug-C60 accumulation

The radius of gyration (Rg) is a factor that enables us to analyze the aggregation and stability of molecules such as polymers and resizing of biological macromolecules such as proteins over time [63]. The average of the gyration radius at initial and final time is shown in Tables 1 and 2. As shown in Table 1, the gyration radius indicates the accumulation of molecules in one region. The low Rg indicated a high accumulation in the location. The Gyration radius of DOX and PAX is about 3 nm, indicating the aggregation radius of drug accumulation on the C60 surface. Due to C60 and simulation boxes' dimensions, a useful aggregation of drugs is formed around the C60. This revealed that the polymer molecules are clustered together in this simulation. PAX also has a lower radius than DOX, indicating a better accumulation of DOX than PAX. Complexation due to the accumulation of PAX molecules is more stable and concentrated. The interaction of hydrophilic polymer DMAA with water molecules and C60 helps to coat the nanocarrier better in the bloodstream, which can improve the hydrophilicity of PAX. According to Tables 1 and 2, the same drugs' accumulation is similar in two different pHs states at the initial time.

**Table 1:** Radius of gyration at neutral pH

	Doxorubicin (nm)	Paclitaxel (nm)	DMAA-TMC (nm)
Initial Rg	3.34	3.46	3.61
Final Rg	2. 08	2.43	2.67

**Table 2:** Radius of gyration at acidic pH

	Doxorubicin (nm)	Paclitaxel (nm)	DMAA-TMC (nm)
Initial Rg	3.46	3.57	3.72
Final Rg	3. 74	3.69	3.91

On the other hand, the higher the gyration radius, the greater dispersion between the particles. As shown in Table 2, At acidic pH, the Rg increase, the stability and aggregation of systems decrease, and the system disassembled. Hence, the release of drugs facilitated at the cancerous acidic microenvironment in comparison with neutral healthy tissues [64].

**Fig. 3:**
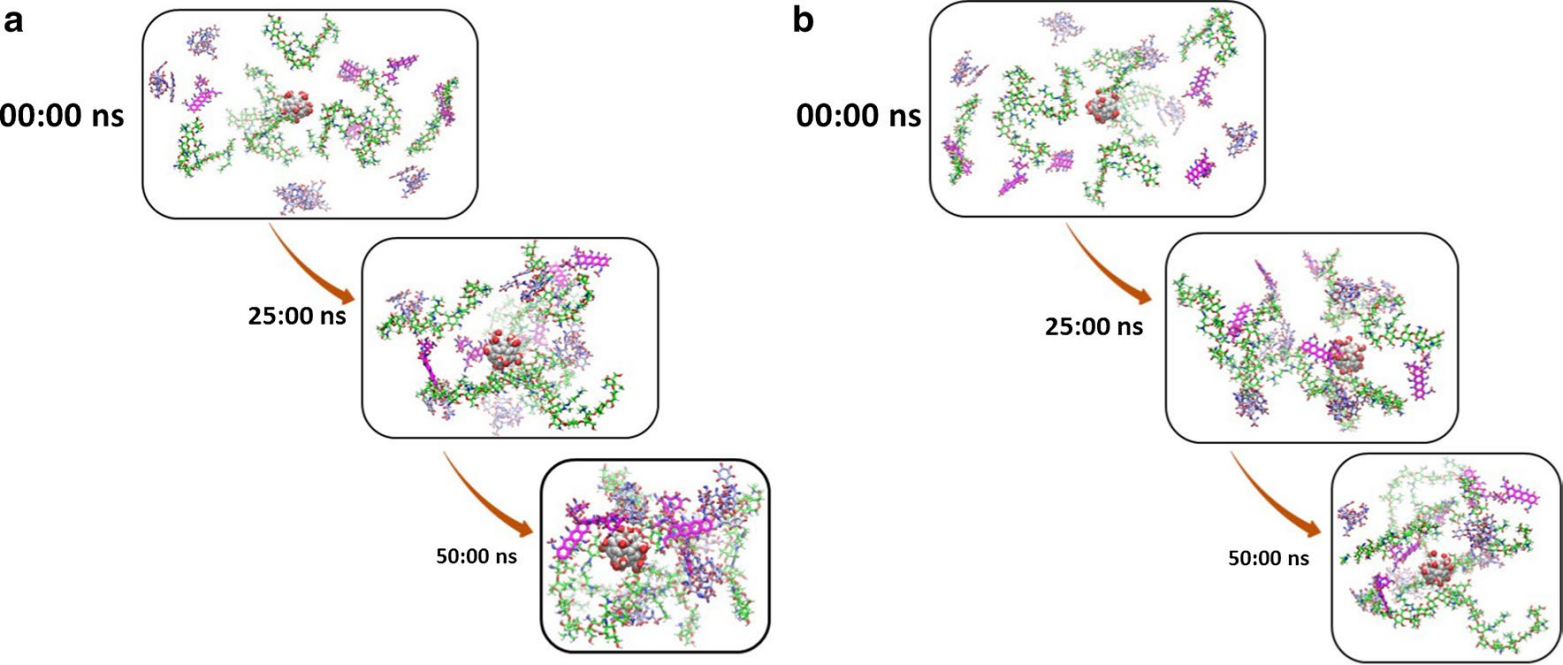
Molecular 3-D figures at 0 ns, 25 ns, and 50 ns of simulation. **a**, **b** are for neutral and acidic conditions, respectively. The high-resolution 3-D figures are given in Additional files 1, 2, 3, 4, 5 and 6

During the simulation, the particle size and Rg fluctuation as shown in Fig. 3 the locations of molecules are different in both pHs at the 50 ns. For studying at high resolution, the figures are attached in Additional files 1, 2, 3, 4, 5 and 6.

### Drug-nanocarrier interaction

Hydrogen bonding between two atoms is defined as a donor–acceptor pair with an angle between them less than 30 degrees. Table 3 indicate the average of the numbers of hydrogen bonds over time between polymer–polymer and polymer-drug and C60-drug for all two pHs condition. Hydrogen bonding can serve as a hydrophilicity indicator of the carrier. Besides, hydrogen bonding is part of the interatomic forces that can contribute to carrier strength and stability. The analysis of the diagrams and average value shows that DOX is not bounded to C60s but has many hydrogen bonds with DMAA-TMC. This happen illustrates the crucial role of DMAA-TMC in this drug delivery system. DMAA-TMC interacts and bonds with C60, which makes it more hydrophilic. C60s and DOX, as well as PAX, are hydrophobic compounds, and this is a significant drawback for drug carriers; Because hydrophobic compounds aggregate in water and compose large particles disrupting drug delivery and block the bloodstream.

**Table 3:** Average numbers of hydrogen bonds between different particles at two pHs

	DOX-DOX	PAX-PAX	TMC-TMC	C60-C60	DOX-C60	PAX-C60	DOX-TMC	PAX-TMC	PAX-TMC
pH = 7.4	1.4	0.6	5.4	0	0	0	2.40	0.40	0.40
pH = 5.5	0.9	0.3	2.3	0	0.50	0	0.26	0.22	0.22

Furthermore, DMAA-TMC has solved this problem by hydrophilic the complex. The values also show that PAX did not form hydrogen bonds with C60s, while PAX makes many hydrogen bonds with DMAA-TMC, as an essential drug delivery factor. A comparison between PAX and DOX Shows that the DOX-DMAA-TMC hydrogen bonds are more robust than the PAX-DMAA-TMC hydrogen bonds. Therefore, the addition of DMAA-TMC also contributes to better adsorption of DOX as the hydrogen bonds between DOX and DMAA-TMC are relatively stable. The average numbers of hydrogen bonds during the 50 ns simulation are mentioned in Table 3.

### Drug release mechanism

**Fig. 4:**
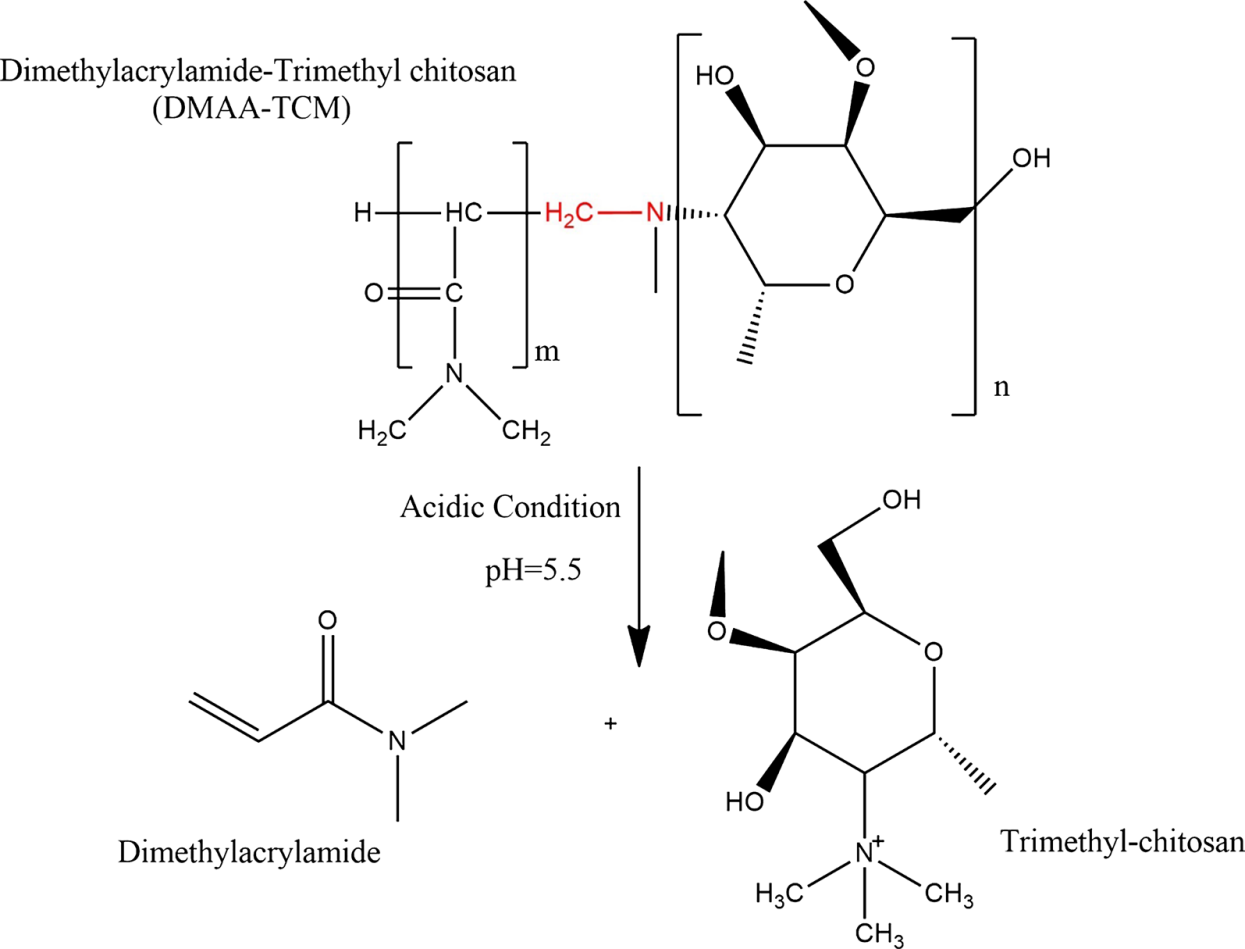
DMAA and TMC copolymer dissimilation mechanism at Acidic pH

As shown in Fig. 4 the Imine group includes a double bond between nitrogen and carbon atoms. The PAX/DOX-loaded nanomaterials via imine linkage could persevere drugs at and disassemble at acidic pH through the cleavage of imine bonds, which would face releasing DOX/PAX immediately [48].

### Quantum calculation

**Fig. 5:**
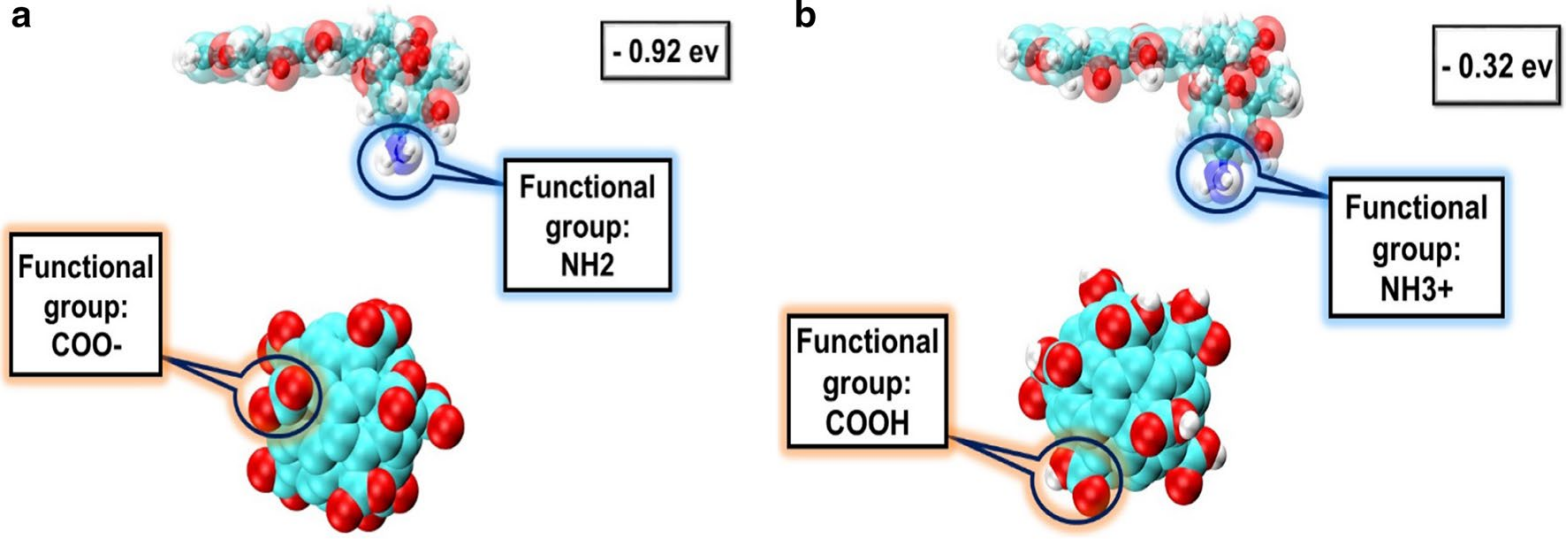
Quantum calculation results. A and B are for neutral and acidic states, respectively

**Fig. 6:**
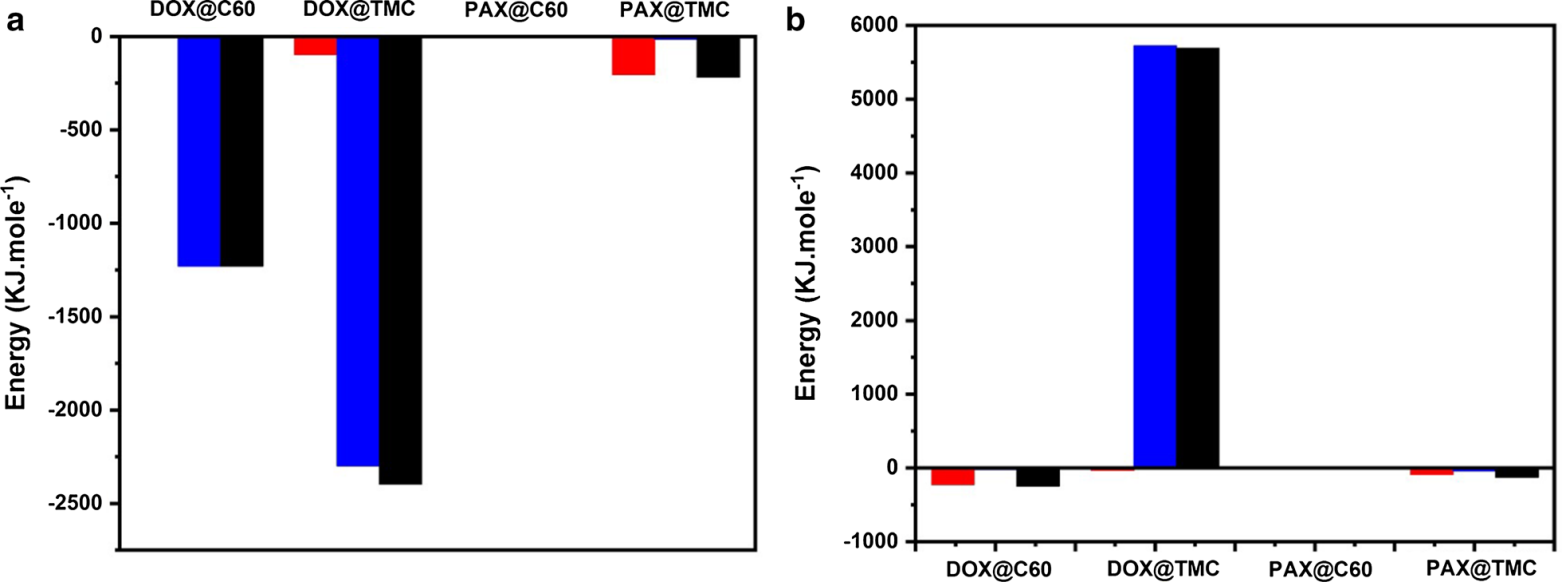
**a** Average energy of DOX/PAX@C60/DMAA-TMC during the 50 ns at pH = 7.4, **b** Average energy of **a**: DOX/PAX@C60/DMAA-TMC during the 50 ns at pH = 5.5

According to Fig. 5, To verifying the study, quantum mechanical modeling is performed by the DFT (Density functional theory) method. Due to the limitations of this method, only the adsorption energy of DOX on C60 was investigated. This value is − 0.32 eV and − 0.92 eV for acidic and neutral state, respectively. According to the results, the absorption energy was more negative in the neutral state (Fig. 5a, b). The results of this calculation confirm our MD simulation results. The functional groups and charges of molecules are shown in the figures (Fig. 6).

### Energy interaction of drug and nanocarrier

#### *Neutral state; pH* = *7.4*

In Fig. 6 A the interaction of the DOX molecule with C60 is investigated. At neutral pH, electrostatic energy plays a significant part in the total interaction energy. While in the acidic state, the electrostatic energy is zero, and the van der Waals energy has a considerable share of the total interaction energy. That is due to the surface charge of the carboxyl functional groups at the C60 surface. The carboxyl group has a negative charge at neutral pH and no charge at acidic pH. On the other hand, DOX has a positive charge at neutral pH and acidic pH. As a result, in the neutral state, drug, and C60 functional groups, have anonymous and robust electrostatic interactions. The higher the electrostatic energy at neutral pH, the higher the drug's adsorption onto the nanocarrier surface at this pH. The critical point is that the drug at a neutral pH, which is the pH of the blood, can transfer satisfactorily to the surface of the C60.

C60, by having a strong attraction to DOX, can serve as an excellent carrier for the drug.

Figure 6 shows the average interaction energy between DOX and DMAA-TMC. As can be seen in part A., the van der Waals interaction is close to zero. Still, there is a significant negative electrostatic interaction between the drug and the polymer. This interaction is due to the positive charge of DOX and the negative charge of the polymer at this pH. The negative electrostatic energy indicates a strong attraction between the drug and the DMAA-TMC. DMAA-TMC is an essential aid in the absorption of the drug.

Part A. in Fig. 6 is the energy interaction of PAX with C60. As shown in the figure at neutral pH, the electrostatic energy shows a more significant number, while the energy van der Waals energy is close to zero. C60 is functionalized with carboxyl functional groups. The carboxyl group has a negative charge at neutral pH and no charge at acidic pH. On the other hand, PAX has zero charges at neutral pH. As a result, the electrostatic energy between PAX and the C60 is close to zero in the neutral state. Van der Waals Energy plays a significant part in the adsorption of PAX onto C60. The following diagram illustrates the energy interaction between PAX and DMAA-TMC at d. As shown in Fig. 6, at neutral pH, Van der Waals energy shows a more significant number, and electrostatic energy is near zero. PAX has zero charges at neutral pH. As a result, the electrostatic energy between PAX and DMAA-TMC is close to zero in the neutral state. Van der Waals energy plays a significant role in the absorption of PAX onto DMAA-TMC.

#### *Cancerous state pH* = *5.5*

In Fig. 6b, the interaction energy of the doxorubicin molecule with C60 is investigated. As shown in the figure at acidic pH, the electrostatic energy is shallow and close to zero. Electrostatic energy is zero at acidic pH, and van der Waals energy significantly contributes to the total interaction energy. This is due to the surface charge of the carboxyl functional groups at the C60 surface. On the other hand, DOX has a positive charge at neutral pH and acidic pH. As a result, in the neutral state, C60 and drug functional groups have nameless charges and find durable electrostatic interactions. However, since C60 and carboxyl group charge became zero at acidic pHs, the electrostatic interaction energy between C60 and DOX is also zero.

Figure 6b indicates the van der Waals and electrostatic energies of PAX and C60. As seen in the figure, the electrostatic energy is close to zero, and the total energy is approximately equivalent to the van der Waals energy. The zero electrostatic energy is due to the zero charge of the carboxyl group at acidic pH. However, the surface charge of paclitaxel is also close to zero. Therefore, the electrostatic interaction between paclitaxel and C60 is zero, and the van der Waals interaction is fragile. The weak interaction energies lead to a better release of the drug from C60 and are considered a decisive factor for the carrier, which can be very useful in drug release

Figure 6 illustrates the interaction average between DOX and DMAA-TMC. The interesting point in the below diagram is that the interaction between the drug and the DMAA-TMC in an acidic state has positive electrostatic energy. This means that there is a repulsion between chitosan and the drug, which is very effective in releasing the drug. Repulsion between DMAA-TMC and the drug causes a better release of the drug from the surface of the DMAA-TMC and C60. In fact, besides biocompatibility and hydrophilicity, DMAA-TMC plays a significant role in the mechanism of drug release in cancer tissue. Also, shows the interaction between DOX and DMAA-TMC. As can be seen from the illustration, the van der Waals interaction is close to zero, but there is a significant negative electrostatic interaction between the drug and the DMAA-TMC. This interaction is due to the positive charge of DOX and the negative charge of DMAA-TMC at this pH. The negative electrostatic energy indicates a strong attraction between the drug and the DMAA-TMC. DMAA-TMC is an essential aid in the absorption of the drug.

Also, Fig. 6b shows the interaction energy between PAX and DMAA-TMC in an acidic state. As can be realized from the picture, the electrostatic interaction with van der Waals is zero between the drug and the chitosan, contributing to drug release. Zero electrostatic energy is due to the zero-surface charge of PAX.

Figure 6a, b shows the average energy during the simulation. In this figure, the average Van der Waals energy is shown in red, the average electrostatic energy in blue, and the average total energy in black. For finalizing the analysis and energies the Gibbs free energy was calculated. This factor is important especially for PAX interactions because of its intrinsic properties and kind of energies.

### Validation tests and Gibbs free energy

To validate the computational procedure, a relevant recent article was chosen to reproduce a part of Gibbs free energy (∆G) calculation [58]. This article has studied the co-delivery of DOX/PAX by a nanotube-chitosan carrier. The average value of ∆G is − 20.75 kcal/mol in the reference work for DOX adsorption on chitosan-nanotube (in acidic pH). The umbrella sampling simulation in GROMACS software was used to reproduce these data. Simulation results yielded a ∆G value of − 21.64 kcal/mol near that of the reference study. These measurements can provide proof of this work and show consistency with previous works [58].

At the end of the previous analyses, Gibbs free energy (∆G) was performed according to the umbrella sampling method. ∆G represents the sum of all the above energies and analyses. The more negative is, the more spontaneous the process. This value computed − 3. 95 kJ mole^−1^ and − 18.54 kJ mole^−1^ for acidic and neutral states, respectively. ∆G at acidic state is close to zero while ∆G at neutral state is more negative. More negative ∆G at neutral mode indicates that this mode is more stable of DDS. All aditional files of the results, including numberical data of simulation have been attached at additional files 1, 2, 3, 4, 5, 6, 7, 8, 9, 10, 11, 12, 13, 14, 15, 16, 17, 18, 19, 20, 21, 22, 23, 24, 25, 26, 27, 28, 29, 30, 31 and 32.

## Conclusion

Combining biomaterials with nanoparticles is one of the essential functions of the smart drug delivery system. The results showed that finding new carriers with new compounds could be useful in improving the pharmacokinetics, pharmacodynamics properties, and therapeutic processes of drugs. This computational study showed that C60 could be a suitable carrier for pH-sensitive smart drug delivery by a pH modification mechanism. We found that the drug is highly absorbed in neutral pH (as blood), and its desired release occurs at acidic pH (like cancerous tissue).

Concerning the effect of trimethyl chitosan, the results show that the combination of C60s and DMAA-TMC is effective in simultaneous absorption and release of DOX and PAX. The previous simulations in similar articles were repeated as a validation test. As a suggestion for future work, this system can be tested in the laboratory environment and living organisms' tissues.

## Supplementary Information

**Additional file 1.** 3-D figure of the system at 0ns and pH = 5.5

**Additional file 2.** 3-D figure of the system at 25ns and pH = 5.5

**Additional file 3.** 3-D figure of the system at 50ns and pH = 5.5

**Additional file 4.** 3-D figure of the system at 0ns and pH = 7.4

**Additional file 5.** 3-D figure of the system at 25ns and pH = 7.4

**Additional file 6.** 3-D figure of the system at 50ns and pH = 7.4

**Additional file 7.** Energy data between DOX and C60 at pH = 5.5

**Additional file 8.** Energy data between DOX and DMAA-TMC at pH = 5.5

**Additional file 9.** Energy data between PAX and C60 at pH = 5.5

**Additional file 10.** Energy data between PAX and DMAA-TMC at pH = 5.5

**Additional file 11.** Radius of gyration of DOX at pH = 5.5

**Additional file 12.** Radius of gyration of PAX at pH = 5.5

**Additional file 13.** Hydrogen bonds between DOX and C60 at pH = 5.5

**Additional file 14.** Hydrogen bonds between DOX and DMAA-TMC at pH = 5.5

**Additional file 15.** Hydrogen bonds between PAX and C60 at pH = 5.5

**Additional file 16.** Hydrogen bonds between PAX and DMAA-TMC at pH = 5.5

**Additional file 17.** RDF of PAX-C60 at pH = 5.5

**Additional file 18.** MSD of DOX at pH = 5.5

**Additional file 19.** RDF of DOX-C60 at pH = 5.5

**Additional file 20.** Energy data between DOX and C60 at pH = 7.4

**Additional file 21.** Energy data between DOX and DMAA-TMC at pH = 7.4

**Additional file 22.** Energy data between PAX and C60 at pH = 7.4

**Additional file 23.** Energy data between PAX and DMAA-TMC at pH = 7.4

**Additional file 24.** Radius of gyration of DOX at pH = 7.4

**Additional file 25.** Radius of gyration of PAX at pH = 7.4

**Additional file 26.** Hydrogen bonds between DOX and C60 at pH = 7.4

**Additional file 27.** Hydrogen bonds between DOX and DMAA-TMC at pH = 7.4

**Additional file 28.** Hydrogen bonds between PAX and C60 at pH = 7.4

**Additional file 29.** Hydrogen bonds between PAX and DMAA-TMC at pH = 7.4

**Additional file 30.** MSD of DOX at pH = 7.4

**Additional file 31.** RDF of PAX-C60 at pH = 7.4

**Additional file 32.** RDF of DOX-C60 at pH = 7.4

## Data Availability

Not applicable.

## Abbreviations

C60: Fullerene
CML: Chronic myelogenous leukemia
DDS: Drug delivery system
DFT: Density functional theory
DMAA-TMC: Dimethyl acryl amid-trimethyl chitosan
DOX: Doxorubicin
GROMACS: Groningen machine for chemical simulations
OPLS*-*aa: Optimized potentials for liquid simulations-all atom
PAX: Paclitaxel
RDF: Radial distribution functions
Rg: Radius of gyration
RMSD: Root mean square deviation
SASA: Solvent accessible surface areas
