# Supplementary figures and images for: Novel pH-responsive nanohybrid for simultaneous delivery of doxorubicin and paclitaxel: an in-silico insight
Source: BMC Chem. 2021 Feb 11;15(1):11. doi: 10.1186/s13065-021-00735-4 (PMC7879683; doi:10.1186/s13065-021-00735-4)

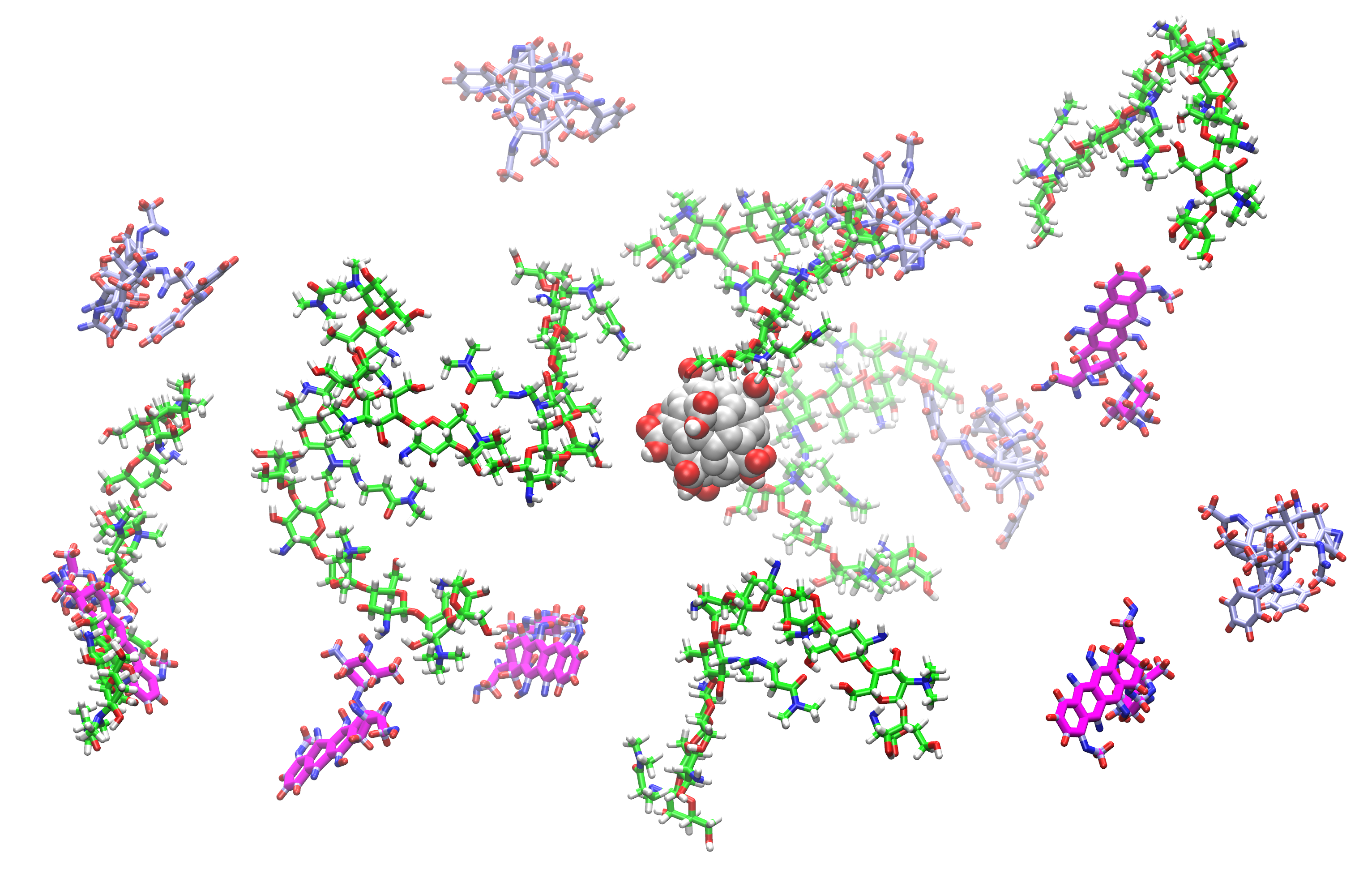

Supplement: Supplementary file 1 — Additional file 1. 3-D figure of the system at 0ns and pH = 5.5 [file 13065_2021_735_MOESM1_ESM.bmp]

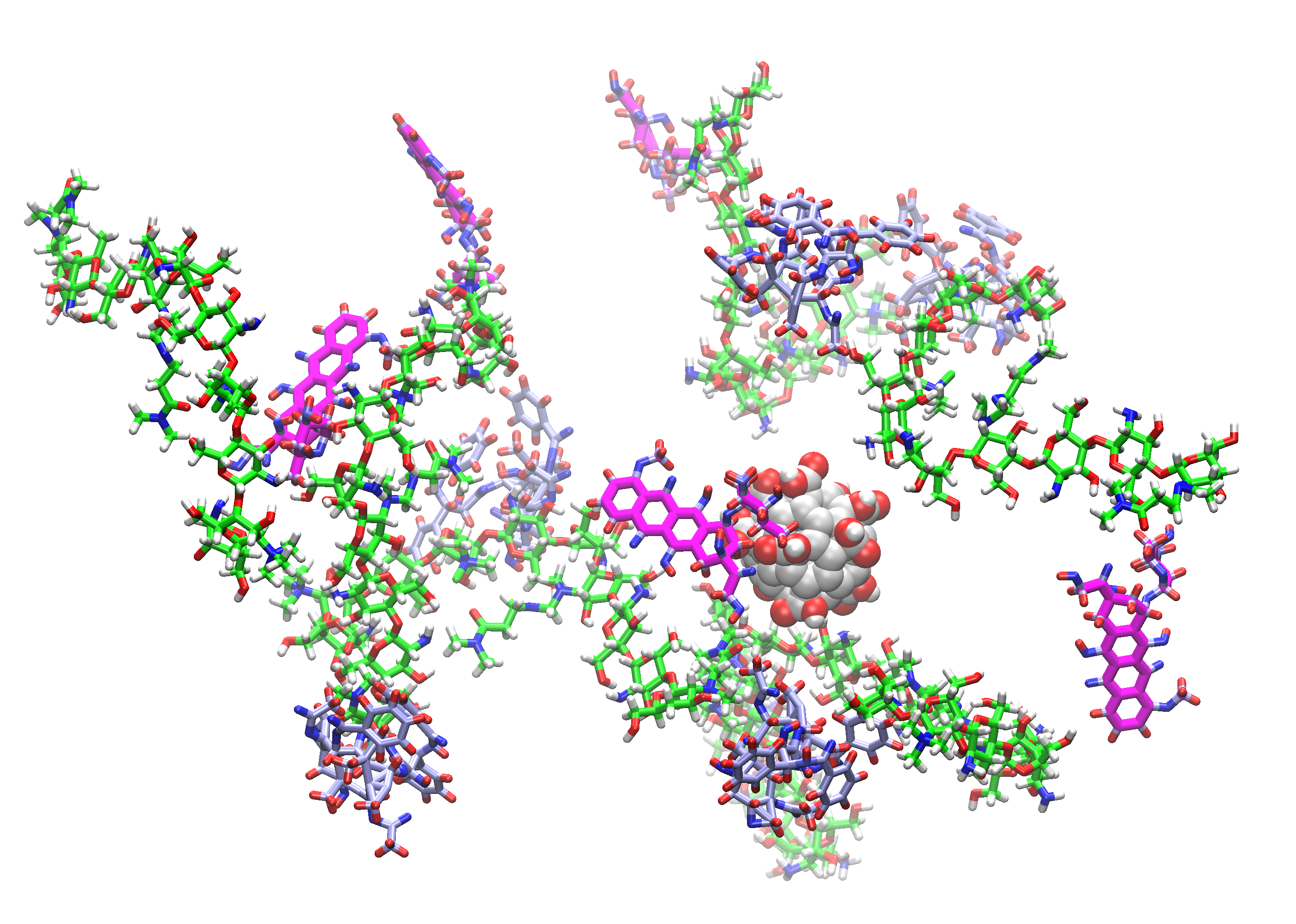

Supplement: Supplementary file 2 — Additional file 2. 3-D figure of the system at 25ns and pH = 5.5 [file 13065_2021_735_MOESM2_ESM.bmp]

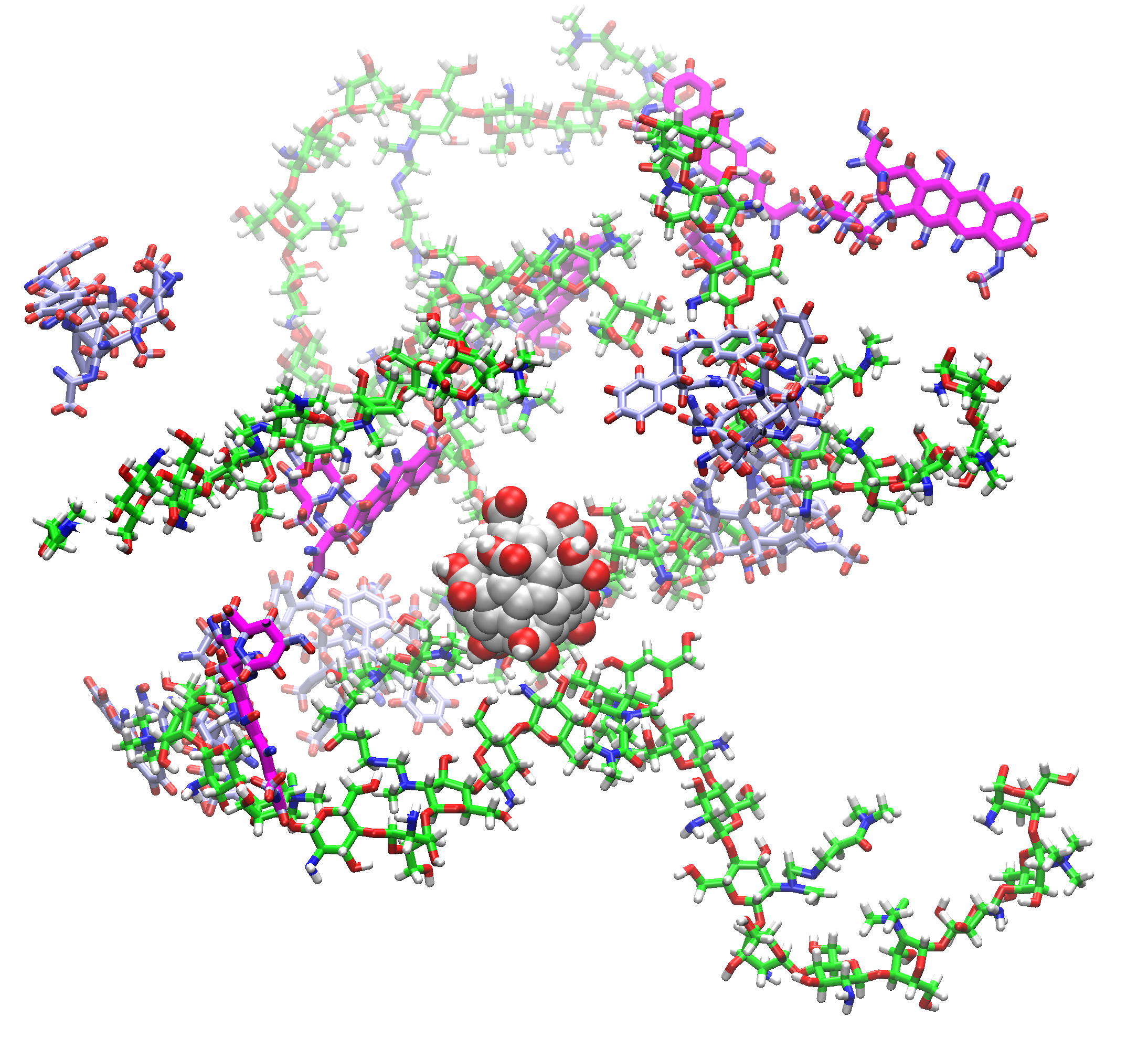

Supplement: Supplementary file 3 — Additional file 3. 3-D figure of the system at 50ns and pH = 5.5 [file 13065_2021_735_MOESM3_ESM.bmp]

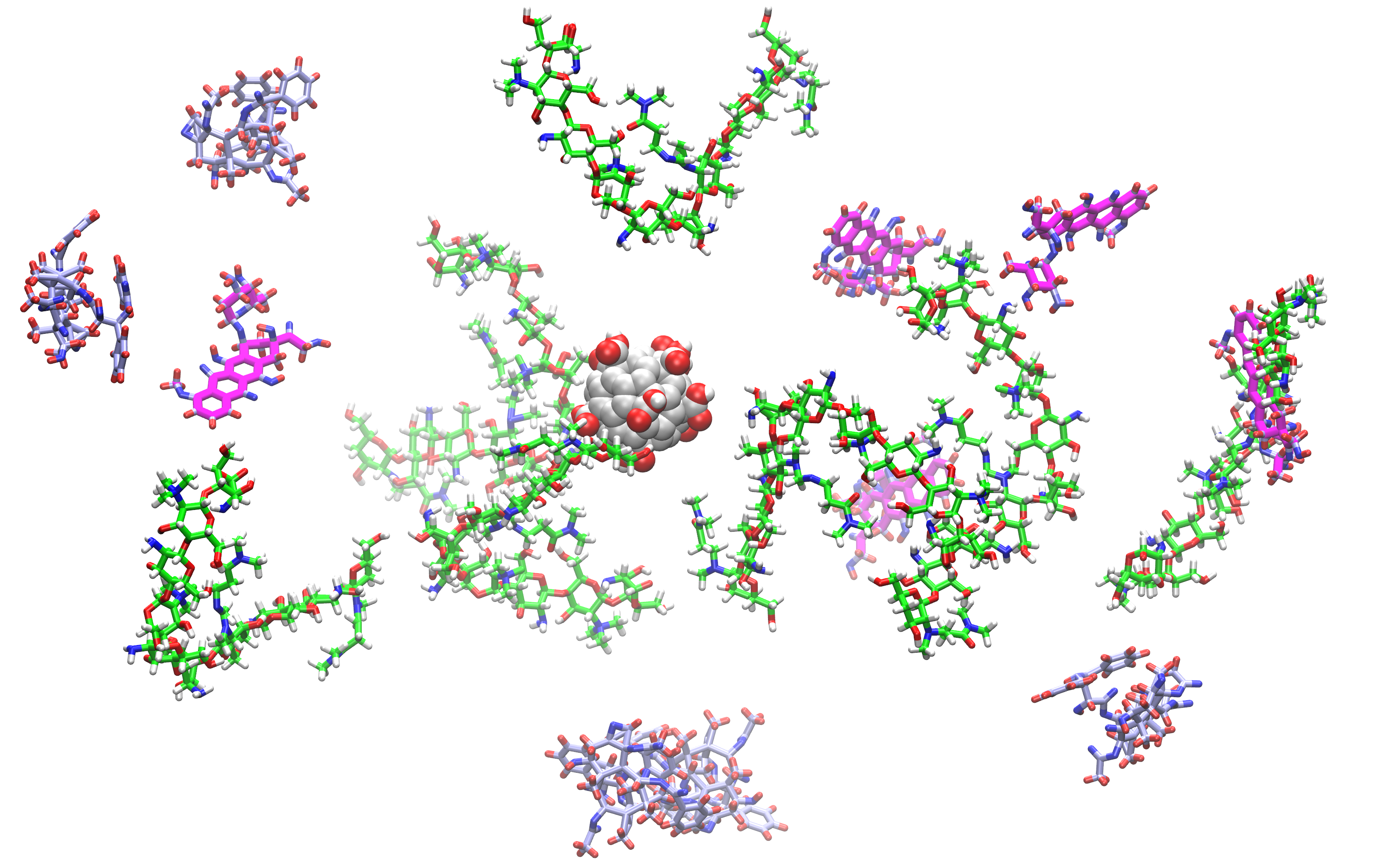

Supplement: Supplementary file 4 — Additional file 4. 3-D figure of the system at 0ns and pH = 7.4 [file 13065_2021_735_MOESM4_ESM.bmp]

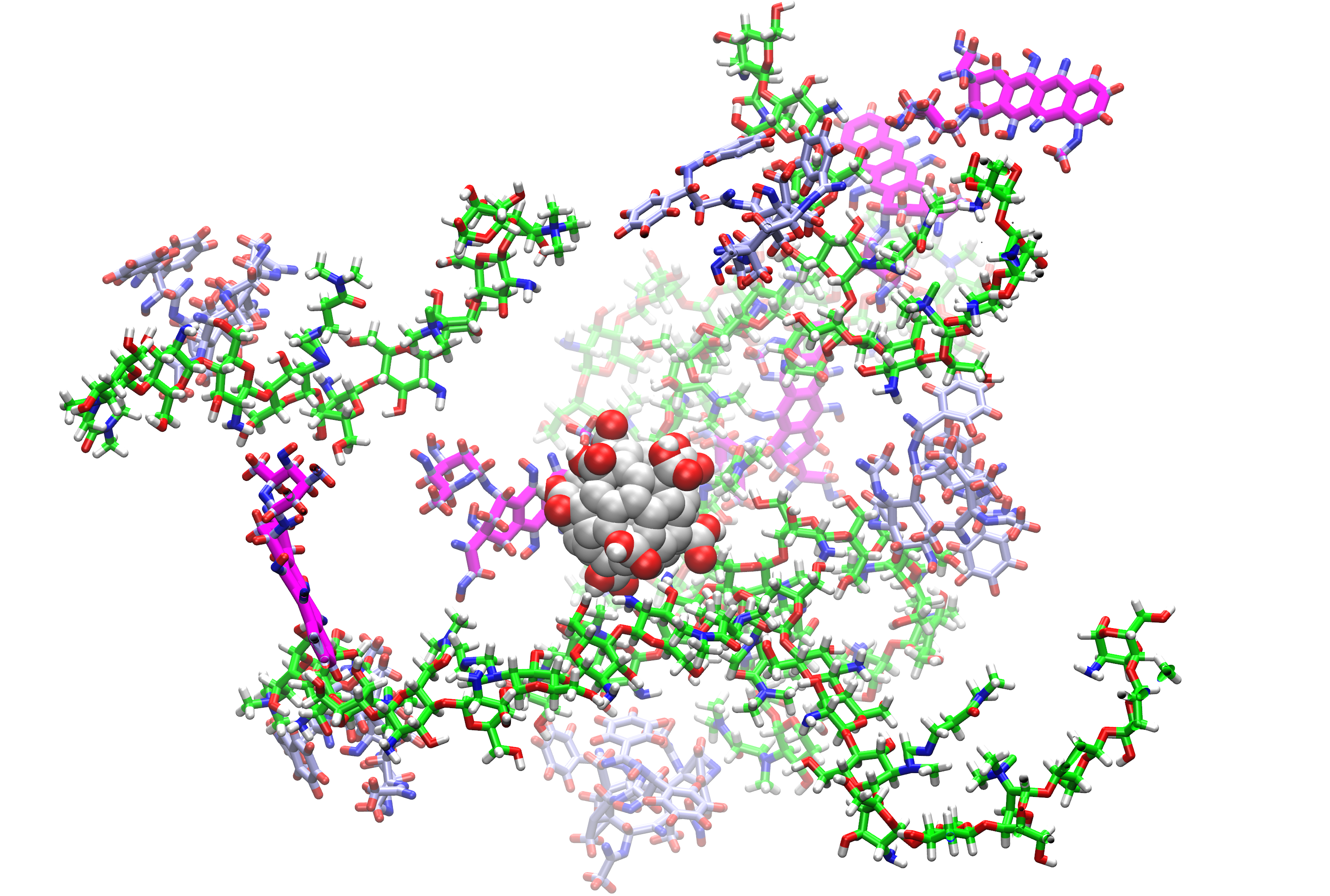

Supplement: Supplementary file 5 — Additional file 5. 3-D figure of the system at 25ns and pH = 7.4 [file 13065_2021_735_MOESM5_ESM.bmp]

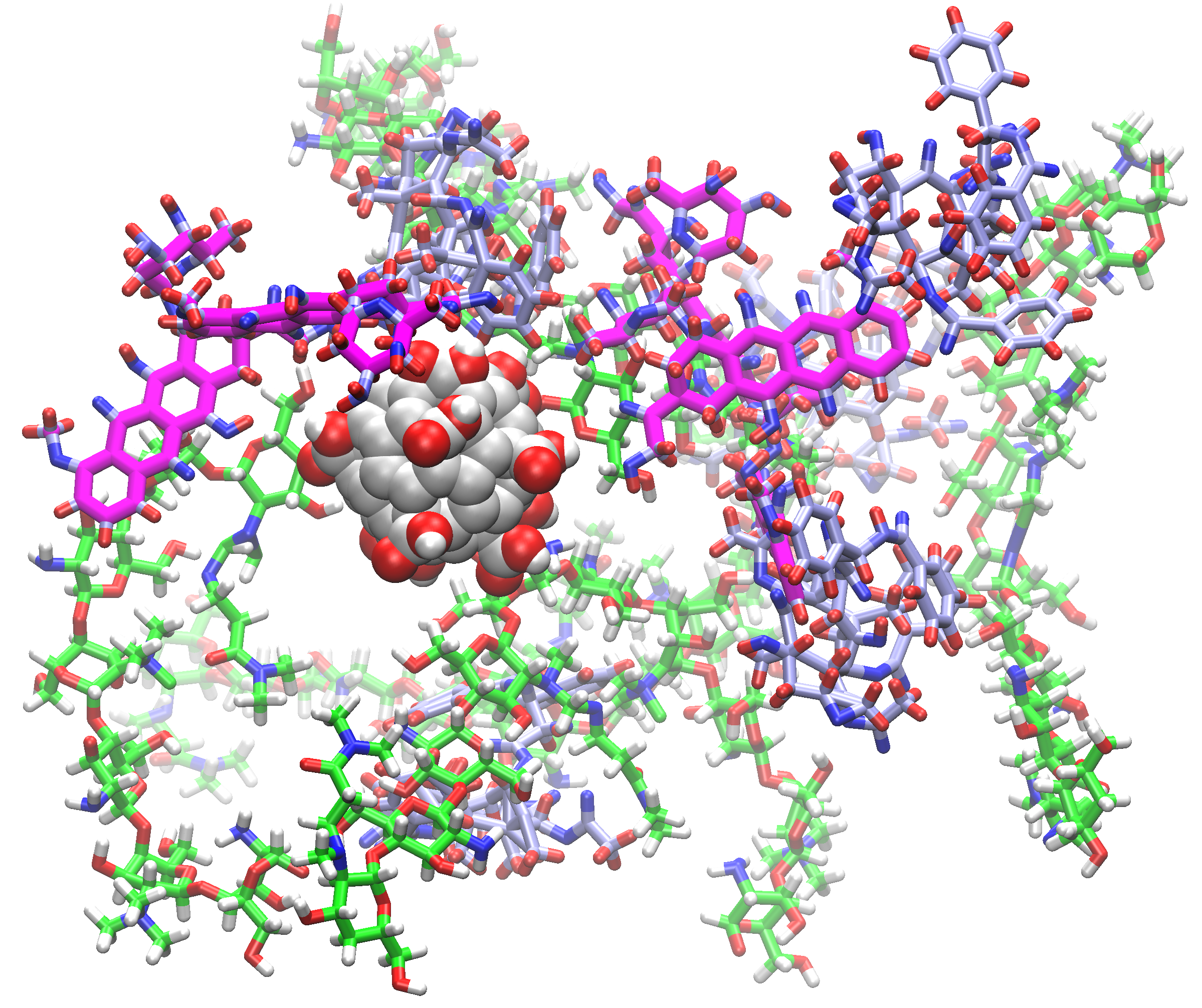

Supplement: Supplementary file 6 — Additional file 6. 3-D figure of the system at 50ns and pH = 7.4 [file 13065_2021_735_MOESM6_ESM.bmp]
